# Emerging role of mesenchymal stem cell-derived extracellular vesicles in periodontal regeneration

**DOI:** 10.1016/j.jtumed.2024.01.006

**Published:** 2024-02-04

**Authors:** Yaldasadat Anvari, Ahmad Afrashteh, Sajjad Pourkaveh, Samira B. Salek, Lelaw Al-Numan, Sahar Khademnezhad

**Affiliations:** a Department of Dentistry, School of Dentistry, Near East University, Nicosia, Cyprus; b Department of Periodontics, School of Dentistry, Tabriz University of Medical Sciences, Tabriz, Iran; c Department of Oral and Maxillofacial Medicine, School of Dentistry, Tabriz University of Medical Sciences, Tabriz, Iran

**Keywords:** الحويصلات خارج الخلية, الخلايا الجذعية الوسيطة, تجديد اللثة, الحويصلات خارج الخلية المشتقة من الخلايا الجذعية الوسيطة, الطب, التجديدي, Extracellular vesicles, Mesenchymal stem cell-derived extracellular vesicles, Mesenchymal stem cells, Periodontal regeneration, Regenerative medicine

## Abstract

Periodontitis is a prevalent oral ailment that harms both hard and soft tissues of the periodontium, leading to loosening and eventual removal of the teeth. Current clinical treatments have limitations in achieving complete periodontal tissue regeneration. Mesenchymal stem cells (MSCs) have garnered attention due to their unique characteristics and potential as a promising new therapy for periodontitis. Research suggests that the role of MSCs in regenerative medicine primarily occurs through the paracrine pathway, involving the emission of particles encased by lipids called extracellular vesicles (EVs) abundant in bioactive compounds. These EVs play a vital function in controlling the activities of periodontal tissues and immune system cells, and by influencing the immediate surrounding, thus fostering the healing of periodontal damage and renewal of tissues. EVs obtained from MSCs (MSC-EVs), in the form of a cell-free treatment, offer advantages in terms of stability, reduced immune rejection, and ethical considerations, elevating their potential as a hopeful choice for broad clinical applications. This concise overview highlights the mechanisms of MSC-EVs and the possibilities they hold in clinical application for periodontal regeneration.

## الملخص

التهاب اللثة هو مرض فموي منتشر يضر بالأنسجة الصلبة والناعمة في اللثة، مما يؤدي إلى تخفيف الأسنان وإزالتها في نهاية المطاف. العلاجات السريرية الحالية لها حدود في تحقيق تجديد كامل لأنسجة اللثة. لقد جذبت الخلايا الجذعية الوسيطة الاهتمام بسبب خصائصها الفريدة وإمكاناتها كعلاج جديد واعد لالتهاب اللثة. تشير الأبحاث إلى أن دور الخلايا الجذعية الوسيطة في الطب التجديدي يحدث في المقام الأول من خلال مسار نظير الصماوي، والذي يتضمن إطلاق جزيئات محاطة بالدهون تسمى الحويصلات خارج الخلية المتوفرة بكثرة في المركبات النشطة بيولوجيا. تلعب هذه الحويصلات خارج الخلية وظيفة حاسمة في التحكم في أنشطة أنسجة اللثة، وخلايا الجهاز المناعي، ومن خلال التأثير على المحيط المباشر، وبالتالي تعزيز شفاء تلف اللثة وتجديد الأنسجة. توفر الحويصلات خارج الخلية التي يتم الحصول عليها من الخلايا الجذعية الوسيطة، في شكل علاج دون استخدام الخلايا، مزايا من حيث الاستقرار، وتقليل الرفض المناعي، والاعتبارات الأخلاقية، مما يزيد من إمكاناتها كخيار متفائل للتطبيقات السريرية واسعة النطاق. تسلط هذه النظرة الموجزة الضوء على الحويصلات خارج الخلية التي تم الحصول عليها من آليات الخلايا الجذعية الوسيطة وتطبيقها السريري المحتمل في تجديد اللثة.

## Introduction

The periodontium is the tooth's supporting structure, which contains soft tissues (i.e., gingiva) and periodontal ligaments (PDLs) and hard tissues (i.e., cementum and alveolar bone). Periodontitis is as an inflammatory illness that results in the deterioration of periodontal tissues, leading to bone resorption and tooth mobility.^1^ Periodontitis occurs as the result of an acute (sometimes aggressive) or chronic inflammation. The disease is closely associated with a fast-paced lifestyle and is influenced by unhygienic behaviors, affecting the internal environment of the oral cavity and leading to the formation of plaque biofilm on dental and gingival surfaces. *Porphyromonas gingivalis*, the primary keystone pathogen in periodontal biofilm, triggers responses from the immune system that result in harm to gingival tissues and bone resorption. Osteoclasts play a crucial role in bone destruction, and bone loss occurs when osteoclast-mediated resorption exceeds osteoblast-driven bone formation. Additionally, the activation of osteoclasts by pathogenic factors from periodontal bacteria, coupled with mechanical stress, contribute to alveolar bone destruction in periodontitis. The principal periodontitis treatments concentrate on plaque elimination and dominating local inflammation including scaling and root planning and surgical treatments for periodontal tissue reconstruction. Those treatments aim to reduce symptoms and halt further disease progression, but fail to treat periodontal attachment loss.^2^, ^3^, ^4^, ^5^ To maintain the functions of teeth and dentition after therapy, some regenerative techniques including guided tissue regeneration, bone grafts, and enamel matrix derivatives are being utilized for 2- to 3-walled alveolar bone deformities.^6^^,^^7^ These techniques rely on mesenchymal stem cells (MSCs) residing in the periodontium, space procurement for the differentiation of MSCs, and accessibility of growth factors. Moreover, circulating MSCs can also aid in the regeneration of periodontal tissues. Nonetheless, the clinical results of those methods are uncertain and unforeseeable. Thus, it is important to improve the various regenerative approaches to retain the forms and functions of the periodontium.

Extracellular vesicles (EVs) are double-layered lipid membrane vesicles that are unable to replicate and include nucleic acids, proteins, lipids, and various messenger molecules.^8^ The majority of eukaryotic cells producing EVs play crucial roles in the transfer of information between cells by mediating communication, which can affect the function of neighboring or other receiving cells.^9^^,^^10^ The paracrine effects exerted by MSCs are managed by EVs, cellular growth regulators, and survival signals. Current research has revealed the advantageous cooperation of MSC-derived EVs (MSC-EVs) in regulating the physiological activities of MSCs.^11^ Due to the challenges of stem cell therapy, recent investigations have focused on additional innovative regenerative techniques including cell-free therapies based on paracrine signaling and utilizing similar produced particles to overcome these hurdles.^12^, ^13^, ^14^, ^15^, ^16^ The studies on stem cells and their mechanisms have shown the substantial function of bioactive compounds within these cells and the so-called conditioned media (CM) adjacent to them. One of the most important secreted molecules released in the biological fluid are EVs that show the same regenerative function as stem cells and thus have the potential to serve as alternatives.^17^ The benefits of EVs compared to stem cell-based treatments are their conservation, sterility, and the possibility of prolonged storage without losing their characteristics. These cell-produced small entities have extensive cell communication activities for various selected cell varieties.

Oral tissues are considered an appropriate source of MSCs, and the first odontogenic stem cells were isolated from a dental pulp in 2000.^18^ Odontogenic stem cells are considered to be an attainable and appropriate source of stem cells with a popular regeneration-promoting capability. Odontogenic stem cells contain numerous varieties including stem cells found in dental pulp mesenchyme, stem cells obtained from shed baby teeth (SHED), stem cells derived from the apical papilla region, stem cells originated from the PDLs (PDLSCs), and progenitor cells from dental follicles.^19^

The aim of this review was to provide an understanding of MSC-EVs based upon the advancing context on periodontal tissues to understand stem cell therapy for periodontal tissue regeneration. In this paper, we first introduced MSC-EVs, as well as their origins, biogenesis, and composition. Then present developments on investigations on their activities in inducing the restoration of periodontal tissue and the various ways they can be applied were summarized. Finally, based on clinical investigation, the possibility of moving scientific findings from the laboratory to practical clinical applications and the difficulties encountered during clinical application were discussed.

## EVs

MSCs, also referred to as mesenchymal stromal cells, exhibit a spindle-shaped morphology. MSCs are multipotent cells capable of differentiating into chondrocytes, osteoblasts, and adipocytes and possess self-renewal capacities.^20^^,^^21^ These cells, obtained from numerous adult tissues,^22^^,^^23^ adhere to tissue culture dishes. Expressed markers on MSCs include cluster of differentiation 73 (CD73), CD90, and CD105; and unexpressed markers include CD45, CD34, CD14 or CD11b, CD79 alpha or CD19, and human leukocyte antigen-DR.^21^ The ethical issues associated with harvesting MSCs are minimal, and they exhibit low immunogenicity,^22^ making them safe and potent cells for stem cell therapy.

Secreted EVs are spherical vesicles with membranes created by a highly heterogeneous double-layered lipid. Their molecular measurements range from 30 to 5000 nm.^24^ Until the late 1990s, EVs were believed to eliminate metabolic waste from cells. Later, the biological activity of EVs garnered interest from various investigators.^25^ MSC-EVs facilitate communication between cells, thereby playing a role in intercellular communication to control the biological activities pertaining to sensory cells, which is considered to have a crucial role in regeneration.

### Biogenesis

EVs cover a wide range of substances, and while there is some size overlap among different EV types, their key difference lies in how they form. Here, we provide a brief overview of the processes for the main three types: exosomes (30–150 nm), formed through endosomes and multivesicular bodies (MVBs); microvesicles (MVs) (100–1000 nm), released directly through cell membrane fusion; and apoptotic bodies (1000 nm–5 μm), generated during cell apoptosis and aiding in cell removal. These processes involve various cellular mechanisms such as endocytosis, vesicle fusion, and apoptosis.

Exosome formation begins with endocytosis. First, during the start of endocytosis, early endosomes form and merge with endocytic vesicles. Later, MVBs are created via two pathways: the endosomal sorting complex required for transport (ESCRT) complex-dependent pathway and the ESCRT complex-independent pathway.^26^, ^27^, ^28^ Inward budding of MVBs results in the formation of intraluminal vesicles, also known as late endosomes, initiating the production of exosomes. MVs are released directly through the fusion and splitting of the cell membrane. Various factors, including the redistribution of phospholipids and shrinking of cytoskeletal proteins, contribute to this process.^29^

**Figure 1:**
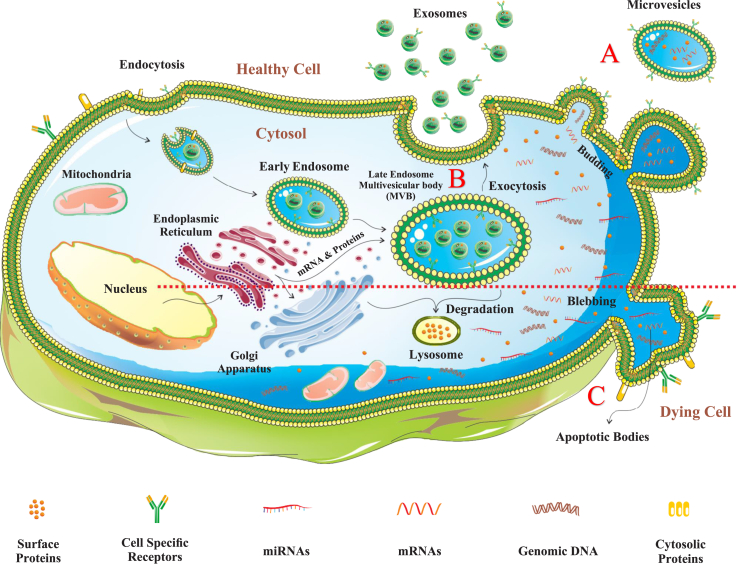
**The processes involved in the maturation and release of EVs.** EVs encompass exosomes, MVs, and apoptotic bodies. A) MVs are discharged through a process of membrane budding. B) Exosomes originate from MVBs and their formation begins with shaping early endosomes as a result of endocytosis. Thereafter, MVBs form late endosomes, which are released as exosomes when MVBs merge with the cell's outer membrane. These EVs transport various cargo such as proteins, lipids, miRNAs, and mRNAs, which are secreted from the endoplasmic reticulum and can be transported to specific target cells. C) In contrast to exosomes and MVs, apoptotic bodies form in a dying cell by means of blebbing.

In contrast to exosomes and MVs, larger apoptotic bodies (ApoBDs), varying in size between 1000 nm and 5 μm, are released when a cell undergoes apoptosis.^30^ Apart from promoting cell interaction, ApoBDs also contribute to the elimination of apoptotic cells (Figure 1).^31^ To summarize, MSC-EVs can be created by various processes and subsequently released from different cells, playing important roles in diverse biological activities including cell-to-cell communication.

### Composition

The diversity of EVs and miscellaneous purifying techniques have led to controversies during investigations of its composition.^32^ By and large, EVs basically include proteins, lipids, DNA, and RNA.^33^^,^^34^ EV biogenesis consists of proteins promoting a relationship between EVs and sensory cells.^35^ Proteins present in MSC-EVs play a crucial role in immune control,^36^ inflammation-associated responses,^37^ and possibly illness control.^38^^,^^39^ The contents of EVs are specific to the cell type, highlighting the characteristics of their parent cells and secretion conditions. Additionally, they contain cell type-independent contents, representing shared features among EVs from various sources. The distinctive EV contents linked to cell types suggest potential therapeutic applications, such as tissue regeneration, and the identification of exosomes as biomarkers for diseases including cancer development or degenerative conditions.^40^^,^^41^

Lipids are rudimentary elements of EV membranes. The distinctions in lipid constitution among EV categories show their miscellaneous biogenesis. Concerning nucleic acids, DNA constitution in EVs is associated with intercell signaling,^42^ cell-based hemostasis,^43^ and genome-based development.^44^^,^^45^ Excluding acting as biomarkers for diagnostic purposes,^46^ according to Eirin et al., by doing RNA sequencing analysis, EV-RNAs can take part in diverse cellular pathways by aiming at transcription elements and genes.^47^ EVs consist of definite motifs due to forming vesicles, from which the vesicles are originated from and the environmental circumstances. Moreover, certain medications can be conveyed in few engineered EVs to be used for therapeutic purposes.^48^ During preliminary stages of MSCs treatment, motifs and activity of their EVs can be influenced. For instance, following lipopolysaccharide (LPS) pretreatment, the paracrine, reconstructive, and regenerative potential of MSCs is notably ameliorated, which has given rise to possible treatments for both inflammatory diseases and tissue wounds.^49^ During periodontal regeneration, EVs produced after exposing dental follicle stem cells (DFSCs) to preconditioning of 250 ng/mL LPS, showed a marked decline in the pathogenesis of *P*. *gingivalis* and a halting reduction in bone density compared to the control group.^50^, ^51^, ^52^ This can be associated with the modified motif of biologically active substances within EVs. According to Zheng et al., PDLSCs undergoing preliminary treatment in the presence of 1 μg/mL LPS showed lower amounts of microRNA-155-5 (miR-155-5) and an increasing response to Sirtuin 1 within the exosomes they generated, which resulted in abatement of the inflammatory microenvironment.^53^

In addition to LPS, MSCs exposed to preliminary TNF-α treatment showed the same results. According to Nakao et al., gingiva-derived MSCs (GMSCs) undergoing preliminary treatment produced exosomes with more miR-1260b, which were capable of inhibiting the expression of Wnt5a mRNA in sensory cells, hence hindering the breakdown of bone tissue.^54^ Furthermore, alterations in MSCs genes play a crucial role in the direct regulation of paracrine secretion. Xu et al.^55^ found that exosomes originating from PDLSCs that have been genetically modified using the P2X7R gene expressed higher levels of miRNA-3679-5, miRNA-6747-5, and microRNA-6515-5 and by attaching to the gremlin 1 (GREM 1) protein, they inhibited inflammation-impaired PDLSC dysfunction. That being said, EV composition not only mirrors the features of their founts but is also inextricable from their activity. By utilizing this characteristic, artificially modifying EVs motifs to induce periodontal regeneration can be a promising research direction.

## MSC-EVs induce periodontal regeneration

**Figure 2:**
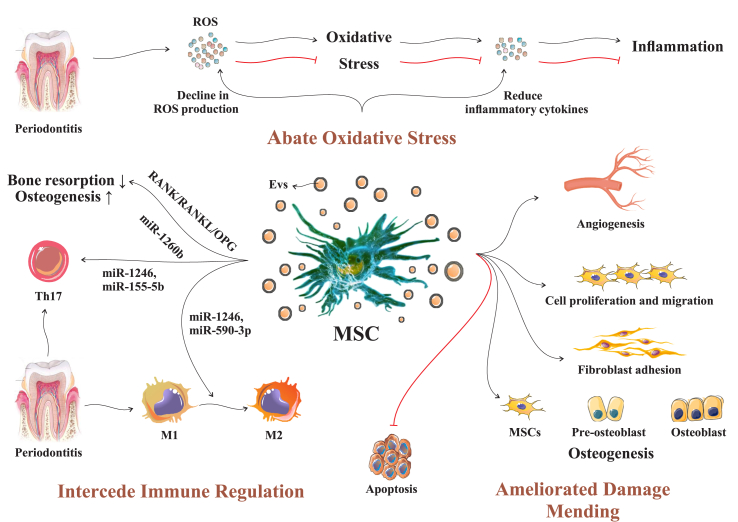
Depiction of how MSC-EVs facilitate the process of periodontal regeneration. MSC-EVs aid periodontal regeneration by countering damage from ROS. They enhance resistance to ROS, promote osteogenesis, and reduce bone resorption. Additionally, these vesicles suppress harmful Th17 cells, transform inflammatory cells, induce angiogenesis, and enhance processes leading to osteoblast formation and osteogenesis. ROS: Reactive oxygen species; MSC: Mesenchymal stem cell; EV: Extracellular vesicle; MSC-EV: Mesenchymal stem cell-derived EV; RANK/RANKL/OPG: Receptor activator of NF-κB/receptor activator of NF-kB ligand/osteoprotegerin; miR-1260b: MicroRNA 1260b; miR-1246: MicroRNA 1246; miR-155-5b: MicroRNA 155-5b; Th17; T helper 17 cell.

MSCs release vectors, such as EVs, which engage in cell-to-cell communication by releasing signaling molecules. This communication moderates information exchange between cells, initiating a chain reaction that affects specific recipient cells and influences the physiological functions of cells in a localized environment. This process promotes the restoration of injured tissues (Figure 2). During the progression of periodontitis, the periodontal tissue can undergo damage from reactive oxygen species (ROS), either directly or indirectly. Direct damage occurs due to the harmful effects of ROS, leading to the oxidative degradation of lipids, proteins, and DNA within cells. Additionally, ROS can have indirect effects, such as triggering inflammation and weakening the immune system. MSC exosomes primarily mitigate free radical damage by enhancing resistance to ROS.

MSC-EVs can induce osteogenesis and a reduction in bone resorption by regulating the receptor activator of nuclear factor kappa B (NF-κB)/receptor activator of NF-kB ligand/osteoprotegerin (RANK/RANKL/OPG) signaling pathway targeted by miR-1260b. During the development of periodontitis, Th17 cells, which are unique among T cells in their ability to harm bone tissue and stimulate the formation of osteoclasts, are suppressed by miR-1246 and miR-155-5b produced by MSC-EVs. The pro-inflammatory phenotype (M1), which is active in periodontitis, is converted to the anti-inflammatory phenotype (M2) to facilitate the regeneration of periodontal tissue while preventing the loss of alveolar bone. The M1 to M2 transformation is stimulated by miR-1246 and miR-590-3p secreted by MSC-EVs. MSC-EVs induce angiogenesis, which is critical for alveolar bone regeneration.

Moreover, MSC-EVs enhance fibroblast adhesion, while also stimulating cell proliferation and migration, which leads to formation of osteoblasts and osteogenesis. Even though the mechanism by which MSC-EVs induce periodontal regeneration is not completely understood, numerous investigations have been conducted on the principal processes (Table 1), which are described below.

**Table 1:** Research on understanding how MSC-EVs contribute to the regeneration of periodontium.

Cell origin	Cell variety	Study design	Agile EV molecules	Processes	Outcomes	Reference
Non-dental derived stem cells	BMSCs	In a controlled laboratory environment	–	Abate oxidative injury	Diminish the inflammation promotion condition of macrophages promoted by LPS-PG	56
		In a controlled laboratory environment and within a living organism (rat model)	–	Facilitate regulation of the immune system	Help with periodontal tissue repair	57
	MSCs	In a controlled laboratory environment and within a living organism (rat model)	–	Ameliorate injury mending	Ameliorate periodontal repair	58
	ADSCs	Within a living organism (rat model)	–		Have immune regulation and inflammation counteracting influences	59
Dental-derived stem cells	PDLSCs	In a controlled laboratory environment	–	Abate oxidative injury	Show inflammation counteracting and restrain immune influences	60
		Within a living organism (murine model) & in a controlled laboratory environment	miR-590-3p	Facilitate regulation of the immune system	Diminish periodontium-related inflammatory damage and pyroptosis of macrophage	61
		In a controlled laboratory environment	miR-155-5p			53
		In a controlled laboratory environment	miR-6515-5p	Ameliorate injury mending	Modify the small-scale environment characterized by inflammation in a specific area and ameliorate tissue repair	55
		Within a living organism (rat model) and in a controlled laboratory environment	–		Help with pro-angiogenesis	62
	GMSCs	Within a living organism (rat model) and in a controlled laboratory environment	–	Abate oxidative injury and conciliate regulation of the immune system	Help with periodontium-related tissue repair	63
		In a controlled laboratory environment	–		Diminish the inflammatory reaction	64, 65
		Within a living organism (mouse model) and in a controlled laboratory environment	exosomal miR-1260b	Facilitate regulation of the immune system	Prevent periodontal bone loss	54
	DPSCs	Within a living organism (mouse model) and in a controlled laboratory environment	miR-1246	Diminish oxidative injury and conciliate regulation of the immune system	Induce alveolar bone and periodontal epithelium mending	66
		Within a living organism (mouse model) and in a controlled laboratory environment	miR-1246	Facilitate regulation of the immune system	Ameliorate inflammation countering feature	67
		In a controlled laboratory environment	miR-378a	Ameliorate injury mending	Ameliorate cell angiogenesis	68
	DFSCs	Within a living organism (beagle dog model) and in a controlled laboratory environment	GSR and SOD1	Diminish oxidative injury and conciliate regulation of the immune system	Prevent reduction in the bone surrounding alveoli and ameliorate periodontium-related repair	51
		Within a living organism (rat model) and in a controlled laboratory environment	–	Ameliorate injury Mending	Leads to alveolar bone repair	69
		Within a living organism (rat model) and in a controlled laboratory environment	–		Induce periodontium-related tissue repair	70
		Within a living organism (rat model) and in a controlled laboratory environment	–		Induce periodontium-related tissue repair within an environment characterized by inflammation	52
	SHED	Within a living organism (mouse model)	–	Diminish oxidative injury and ameliorate injury mending	Ameliorate the osteogenic capability of BMSCs	71
		In a controlled laboratory environment	Wnt3a and BMP2	Ameliorate injury mending	Ameliorate the osteogenic capability of PDLSCs	72

BMSC: Bone marrow-derived mesenchymal stromal cell; MSC: Mesenchymal stem cell, ADSC: Adipose-derived stem cell; PDLSC: Periodontal ligament stem cell; GMSC: Gingival mesenchymal stem cell; DPSC: Dental pulp stem cell; DFSC; Dental follicle stem cell; SHED: Stem cells from human exfoliated deciduous teeth; LPS-PG: Lipopolysaccharide from *Porphyromonas gingivalis*; BMP2: Bone morphogenetic protein 2.

### EVs actively participate in abating oxidative stress injury

Recently, there has been significant attention on periodontal tissue injury resulting from an in equilibrium in the generation of ROS and the body's detoxification capacity.^73^ In the course of periodontitis, periodontal tissue can be directly or indirectly destructed by ROS. Excess ROS can directly damage cells through oxidative breakdown of lipids, proteins, and DNA, both inside and outside cells. Additionally, proteases within the matrix can contribute to the deterioration of the matrix outside cells. Subsidiary influences lead to an inflammatory reaction and immunity network impairment. MSC exosomes reduce free radical damage mostly by ameliorating opposition to ROS. According to Huang et al,^51^ analysis of the order of DFSC-derived small EVs with and without LPS therapy at the protein scale depict that EVs on their own convey antioxidants, counteract ROS directly, and inhibit the ROS/JNK pathway, hence performing a crucial role in antioxidant mechanism. Elevated oxidative stress may induce cytokines that promote inflammation production from cells of the immune system, and intensify the inflammatory reaction. EVs may also diminish the production of cytokines associated with inflammation, such as interleukin 6 (IL-6), IL-1β, interferon gamma (IFN-γ), and tumor necrosis factor alpha (TNF-α), which are produced by immune cells as well as cells in the periodontal tissue.^56^, ^64^, ^65^, ^71^, ^74^

Studies, both in controlled laboratory settings and within living organisms, have revealed that EVs participate in diminishing the activation of nuclear factor kappa B (NF-κB). NF-κB is a pivotal regulator in processes related to inflammation and oxidative stress, and EVs lead to the downregulation of the reaction to inflammation. A study conducted by Čebatariūnienė and colleagues demonstrated that EVs have a lasting inhibitory effect on NF-κB activity in PDLSCs, whether under normal conditions or induced by LPS. The inhibition takes place by increasing the activation of Akt through phosphorylation of its target protein, glycogen synthase kinase-3β, while not impacting the mineralization associated with osteogenesis in PDLSCs.^75^ Furthermore, Shen and collaborators observed that exosomes from dental pulp stem cells (DPSCs), namely miR-1246, downregulate the inflammatory response by inhibiting the signaling pathways involving NF-κB and p38 mitogen-activated protein kinase (MAPK).^76^

### MSC-EVs participate in immune regulation in the periodontium

The immune regulatory activity of MSC-EVs is a main feature in the context of their use in medical practice. EVs from various sources have diverse influences on infectious diseases. In patients with periodontitis, MSCs-EVs take part in immunomodulation by various mechanisms and are able to stimulate the transition of the localized environment where immune cells interact and respond to different stimuli and pathogens within a specific tissue or organ, which can lead to tissue regeneration and restoration. Osteoimmunology, described as the interaction connecting bone cells and the immune network, aids in understanding the development of periodontitis. The RANK/RANKL/OPG pathway plays a vital role in periodontal osteoimmune mechanisms. The pivotal regulator, RANKL, significantly influences osteoclast formation, and its mRNA and protein levels are directly associated with the seriousness of periodontitis in individuals. RANK acts as the receptor for RANKL, while OPG serves as a mimic receptor, inhibiting the attachment of bone to RANK through interacting with RANKL.

Research has confirmed that MSC-EVs play a role in the inflammatory environment by engaging with the RANK/RANKL/OPG pathway, thereby reducing excessive bone resorption induced by osteoclast activation.^51^^,^^57^ In the study conducted by Nakao and colleagues,^54^ GMSC-derived exosomes, treated with TNF-α, led to the upregulation of miR-1260b. This specific microRNA targets the Wnt5a-mediated RANKL pathway, resulting in the inhibition of osteoclastic activity.

The regulation of immune responses related to bone function is closely connected to network of immune cells. Within these networks, macrophages, in addition to various other components within the immune system, have a vital function in the development of periodontitis. Numerous elements have the ability to influence the reversible nature of macrophage (Mø) polarization, resulting in two distinct characteristics: one exhibits pro-inflammatory characteristics (M1), while the other corresponds to anti-inflammatory characteristics (M2), often referred to as the characteristics associated with the process in the process of wound recovery. Research has shown that converting macrophages from the M1 to M2 phenotype promotes the regeneration of periodontal tissue and curbs reduction in alveolar bone density.^77^ MSC-EVs can induce this phenotypic shift in macrophages, transitioning them from M1 to M2, by means of various communication routes in an inflammatory environment.^51^^,^^65^^,^^57^ Shen et al.^66^ discovered that in mice experiencing periodontal diseases, DPSC-exo-incorporated chitosan hydrogel (DPSC-exosome/CS) treatment promoted the change in macrophage state (Mø) towards the M2-type possibly through the involvement of miR-1246 within DPSC-exosomes. Additionally, the mechanism leading to macrophage cell death through pyroptosis, which initiates an inflammatory response, was addressed. As described by Han and colleagues,^61^ hPDLSC-EVs carrying miR-590-3p blocked the genetic expression of TLR4 in macrophages, leading to reduced and subsequent alleviation of inflammatory damage.

Apart from macrophages, the role of T cells in controlling the immune reaction during periodontitis is crucial. CD4+ T cells include common subsets such as T helper 17 (Th17) cells and regulatory T cells (Treg) cells. Th17 cells can promote bone resorption and the crucial formation of osteoclasts necessary for defending the host. Conversely, Treg cells hinder excessive or prolonged reactions by the immune system, effectively inhibiting osteoclast formation.^78^ In studies involving mice and canine subjects, inducing the accumulation of Treg cells in periodontal tissue has been shown to suppress the development of periodontitis.^79^ MSC-EVs have been proven to rejuvenate irritated periodontal tissue in both living subjects and controlled experiments.^74^ Researchers have identified key active molecules, such as miR-1246 found in exosomes from DPSC-3D^67^ and miR-155-5p found within exosomes of PDLSC origin,^53^ which play a role in this procedure by suppressing the presence of nuclear factor of activated T cells 5 (Nfat5) in cells and influencing the sequential polarization of reactive Th17 cells, with the former exerting its influence following a specific order. Meanwhile the latter downregulates Th17 cells via the miR-155-5p/sirtuin 1 (SIRT1) pathway, leading to improved anti-inflammatory properties.

In short, the immune regulatory activity of MSC-EVs is a prominent feature in medical applications, particularly in addressing infectious ailments. In the context of periodontitis, MSC-EVs participate in immunomodulation, influencing the localized environment where immune cells respond to stimuli, contributing to tissue healing. The RANK/RANKL/OPG pathway plays a crucial role in periodontal osteoimmune processes, and MSC-EVs engage with this pathway, reducing overly excessive removal of bone induced through the activation of osteoclasts. MSC-EVs induce a shift in macrophage polarization from M1 to M2, promoting periodontal tissue regeneration. Additionally, they impact T cells, influencing the balance between Th17 cells and Treg cells, contributing to the suppression of periodontitis development. Key molecules within MSC-EVs, such as miR-1246 and miR-155-5p, play roles in suppressing Nfat5, influencing Th17 cell polarization and downregulating Th17 cells via the miR-155-5p/SIRT1 pathway, enhancing anti-inflammatory properties. Overall, MSC-EVs exhibit therapeutic potential in rejuvenating inflamed periodontal tissue.

### MSCs ameliorate periodontal tissue regeneration

Vesicles from outside the cells that are discharged by MSCs convey substances with a physiological impact on living organisms and communicate with cells surrounding the damaged tissue region, which can impede their ability to stimulate bone formation. Exosomes originated from stem cells obtained from SHED have ameliorated capability to stimulate the bone formation of periodontium-related tissue cells. According to Wei et al.,^71^ SHED-exosomes are also able of preventing bone marrow-derived MSCs (BMSC) apoptosis and abate their adipogenicity in *in vivo* investigations. Additionally, according to Wang et al.,^72^ Wnt3a and bone morphogenetic protein 2 (BMP2) present within exosomes released by stem cells from exfoliated deciduous teeth (SHED-exosomes) stimulate the BMP/Smad and Wnt/β-catenin pathways. Subsequently, the ability of PDLSCs to stimulate bone formation is enhanced by increasing the phosphorylation of Smad1/5/8 and nuclear β-catenin protein.

According to Xu et al.,^80^ in exosomes produced by PDLSCs after alteration of P2X7R, miR-6515-5 is upregulated. MiR-6515-5 prevented the gene expression of GREM1 that is specifically affected by the transforming growth factor (TGF)/BMP pathway, hence notably inducing the process by which cells develop into bone-forming cells, leading to the formation of bone tissue. Similarly, according to Yi et al.,^69^ *in vitro* investigations have shown that EVs are capable of inducing DFSCs to stimulate bone formation by stimulating phospholipase C (PLC), protein kinase C (PKC), and MAPK. The procedure for regenerating periodontal tissue is complex because periodontal injuries heal on avascular and non-vital hard tissues. In addition to being linked with the connection of a sequence of osteogenic-associated molecules (including signaling proteins such as BMP9, TGF-1, RUNX2, and osteocalcin) to essential components such as calcium, phosphorus, and collagen molecules within the extracellular matrix, alveolar bone needs a considerable extent of angiogenesis to make sure there is a sufficient blood supply.

Abnormal angiogenesis occurs when periodontitis leads to progressive inflammation and EVs are capable of ameliorating the vascular activity of recently created PDLs under these circumstances. According to Zhou et al.,^68^ when a laboratory-based model including DPSCs with periodontal defects was designed, endothelial cell-mediated angiogenesis was stimulated by the Hedgehog/Gli1 communication pathway activation and suppressor of fused (Sufu) downregulation. One of the substantial angiogenesis regulators is vascular endothelial growth factor (VEGF), which stimulates the endurance, expansion, and relocation of cells that form the inner lining of blood vessels.^81^ According to an investigation by Zhang et al.,^62^ angiogenesis using human umbilical vein endothelial cells (HUVECs) was induced by the production of exosomes by PDLSCs. HUVEC angiogenesis was also stimulated as a result of upregulating VEGFA, which has a crucial role in communication between these two cell types. This occurs as a result of inflammation of the periodontal tissue microenvironment preventing, the upregulation of miR-17-5p in PDLSCs and reducing VEGFA, leading to superior conveyance of VEGFA. Abnormally expressed VEGFA is transported by exosomes from PDLSCs to HUVECs, stimulating their development, and hence the amelioration of PDL angiogenesis.

The essential role of cells within the periodontal tissue is pivotal in the process of restoration and healing of periodontal tissue. In their study, Yi et al.^69^ obtained collagenase-released MVs (CRMVs) from a solution of DFSC digested with collagenase. These CRMVs successfully enhanced DFSC proliferation, migration, and their ability to undergo osteogenic differentiation *in vitro*. These effects may involve the PLC/PKC/MAPK pathway.^58^ Furthermore, EVs also play a role in promoting the adhesion and migration of fibroblasts, which in turn contribute to the formation of new PDL attachments. For example, adipose-derived stem cell (ADSC)-exosomes have been shown to stimulate fibroblast movement, growth, and various alternative biological activities, as well as support endothelial angiogenesis in a rodent research model.^59^

In brief, MSC-EVs, particularly exosomes from stem cells obtained from SHED, exhibit therapeutic potential in stimulating bone formation and preventing apoptosis in periodontium-related tissue cells. These exosomes enhance the phosphorylation of signaling proteins involved in bone formation pathways. Moreover, EVs contribute to complex processes such as angiogenesis, with Hedgehog/Gli1 pathway activation and Sufu downregulation inducing endothelial cell-mediated angiogenesis. VEGFA has a pivotal function in this context, and exosomes from PDLSCs contribute to angiogenesis by transporting VEGFA. Additionally, CRMVs from DFSCs enhance cell proliferation, migration, and the osteogenic differentiation process are influenced by the PLC/PKC/MAPK pathways. EVs, including adipose-derived stem cell exosomes, promote fibroblast activities and endothelial angiogenesis, suggesting their role in forming new PDL attachments. Overall, these findings highlight the multifaceted contributions of MSC-EVs in the regeneration of periodontal tissue and healing.

In conclusion, the paragraphs emphasize the potential for therapeutic applications of MSC-EVs in addressing periodontal issues. MSC-EVs play a crucial role in mitigating the damaging effects of periodontitis by alleviating oxidative stress, inhibiting inflammation, and promoting tissue regeneration. Their engagement with the RANK/RANKL/OPG pathway highlights their ability to reduce excessive bone resorption induced by osteoclast activation. Furthermore, MSC-EVs exhibit immunomodulatory effects by influencing macrophage polarization, impacting T cell balance, and suppressing inflammatory responses. Specifically, exosomes from SHED baby teeth and other sources enhance bone formation, prevent apoptosis, and contribute to angiogenesis. The multifaceted contributions of MSC-EVs underscore their potential in fostering periodontal tissue regeneration and healing.

## Application of EVs in clinical studies and their restrictions

Despite the fact that periodontal regeneration stimulating-influence of MSC-EVs on various animal designs have been assessed by preclinical investigations, clinical experiments for periodontitis mending are presently limited. According to a preliminary Phase 1 clinical experiment (NCT04270006, Egypt) administered by Beni-Suef University to evaluate the effect of ADSCs-exosomes on scaling and root planning as a supporting medium for periodontitis treatment. People aged 18–50 years with advanced periodontitis (stage III or IV) were enrolled and individuals without periodontitis were included as controls. Nevertheless, particular characteristics and developments have not been enhanced. The majority of clinical experiments not related to periodontal conditions using MSC-EVs to facilitate specific tissue regeneration are described.^82^ Over a few clinical experiments have been recorded on “www.clinicaltrials.gov*”* on protection and treatment implication of MSC-EVs for further illnesses (Table 2). Amid these, one experiment (NCT04493242, US) has not yet been finalized. In this trial, BMSCs-EVs were applied intravenously to treat COVID-19-related acute respiratory distress syndrome (ARDS). They revealed that BMSC-EVs were harmless and are likely to aid in reinstating oxygenation, suppressing cytokine commotion, and reconstructing immunity, which makes them an promising treatment for severe COVID-19 therapy.^83^

**Table 2:** Clinical experiments with MSC-EVs in periodontitis and different medical conditions.

Disease	Mesenchymal stem sell variety	Nation	NCT number	Stage
**Oral ailments**				
Periodontitis	Adipose-derived stem cells	Egypt	NCT04270006	Not known
**Trauma**				
Burn injuries	Bone marrow-derived mesenchymal stromal cells	Not presented	NCT05078385	In pre-recruitment phase
Injury mending	Adipose-derived stem cells	China	NCT05475418	In pre-recruitment phase
**Newborn-associated ailments**				
Premature infants with precarious bronchopulmonary dysplasia	Bone marrow-derived mesenchymal stromal cells	USA	NCT03857841	Concluded
Newborns with exceedingly low weight at birth	Mesenchymal stem cells	Russia	NCT05490173	In pre-recruitment phase
**Organ transplant**				
Abdominal solid organ transplant	Mesenchymal stem cells	Not presented	NCT05215288	In pre-recruitment phase
**Autoimmune ailment**				
Dystrophic epidermolysis bullosa injuries	Bone marrow-derived mesenchymal stromal cells	Not presented	NCT04173650	In pre-recruitment phase

Several patents related to EV administration in periodontitis therapy supply reinforcing proof for clinical application. Tian et al. innovated two varieties of patents for the treatment of periodontitis and bone defects. In one of the patents invented by Tian et al. (CN202010607634.9), a parenteral medication structure embodying DFSC-originated exosomes was shown to be an excellent periodontal therapy. Another patent discovered by Tian et al. (CN202110910962.0) revealed that MSC-originated ApoBDs can stimulate formation of new bone and aid in the induction of periodontal regeneration, hence playing an imperative role in treating bone defects. Additionally, a calcified collagen gel filled with GMSC-exosomes was invented by Xu et al. (CN202210351949.0). The artificial gel is injectable in the bone defects of patients with periodontitis, and induces local bone regeneration or bone enlargement. According to Jiang et al. (CN202110125452.2), the capability of exosomes from SHEDs to stimulate the rapid growth and bone-forming transformation of BMSCs was confirmed, and it was also verified that they are capable of mending and regeneration of alveolar bone in patients with periodontitis.

In clinical administration, there are numerous benefits of EVs over MSCs, including durability, immune dismissal, and ethical surveillance. MSC-EVs seem to be a promising prospect for periodontal regeneration treatment, but some issues still remain unsolved. To begin with, none of the standard techniques utilized for extraction and characterization investigation are generally approved. MISEV2018^84^ revealed that none of the extracting approaches are ideal. Hence, the source and administration of EVs should be considered when opting for varied approaches. In that instance, setting up standards, industrialization and institutionalize of EVs can be quite challenging. Hence, desegregation of currently comprehended mechanisms is required. Moreover, the challenges associated with clinical administration including safety, forms of administration, dosage, rate of recurrence, drug movement within the body, and adverse reactions still need to be analyzed. Prior to clinical administration, proper verification is required to ensure the safety and efficiency of the treatment, in which a few factors including quantity recognition, size, characteristics, and purity of the EVs should be considered.^85^

## Future prospects of EVs applications

Numerous investigations have focused on biomedical EV administration. Based on their physical activities, in specific varieties of cells, EVs have been utilized as treating moderators in immune treatment, delivering drugs, trials on vaccination, and regenerative medicine. For example, according to Xu et al.,^86^ use of EV for the progress of diagnosing cancer and treatments was summarized. Mianehsaz et al.^87^ assessed the proof for EVs from MSCs as a recent acellular treatment approach for osteoarthritis and joint injury.

EVs from healthy PDLSCs have the potential of enhancing the formation of bone tissue of native stem cells within an environment characterized by inflammation. This is achieved by reinstating the capacity of osteogenic differentiation of inflamed PDLSCs through the inhibition of canonical Wnt signaling.^88^ Moreover, BMSC-EVs present a cell-free strategy for periodontal regeneration, affecting the signaling pathway involving OPG/RANKL/RANK for regulation of osteoclast function. Additionally, they influence polarization of macrophages and the expression of TGF-β1, thereby adjusting the immune response characterized by inflammation and impeding the advancement of periodontitis and damage to the immune system in periodontal tissue.^57^ The upcoming EV generation of products to be investigated and progressed, which are capable of being administered in oral and craniofacial diseases, include artificial EVs, adjustable EVs, and EVs imitating substances along with EVs created to demolish communication pathways related to pathological circumstances.^89^ There needs to be more sufficient standards described regarding isolation, manipulation, and characterization of EVs to achieve future development in clinical administration of EVs. Details including the dissemination, contents, and the refinement process agreements can influence EVs imprints.^90^ Considering the fact that EV cargoes rely notably on their founts, it's imperative to characterize EVs prior to clinical administrations.^91^ Two methods are frequently utilized as purification methods: recapitulated ultracentrifugation or ultrafiltration. These methods solely include EVs with low yield and comparatively take longer time. For instance, solely 5–6 μg of EVs can be delivered by 5 × 106 myeloma cell.^92^, ^93^ Accomplishing proper construction practices demand progress in EV separation methods. Hence, progress in EVs investigations highly depend on discovering new approaches to separate them effectively. It should be acknowledged that administering therapeutic doses of EVs to target cells, especially through systemic injection, can be more complex than it may seem. Riau et al.^94^ suggested the potential use of encapsulated EVs incorporated into biodegradable or highly permeable hydrogels. There have been various studies on techniques focusing on the process of enclosing nanoparticles, such as EVs, within a protective shell or matrix and the exploration of suitable substances to prolong the release of therapeutic agents from stem cells.

Following administration, EVs are dispersed through the bone, lung, spleen, liver, and kidney. Consequently, it becomes essential to evaluate how the organs clear the substance and determine the eventual dose.^95^ The specific endpoint of the physiological impacts induced by EVs on biological systems remains uncertain, even at the time of achieving favorable outcomes. Additionally, the half-life of the applied EVs should be long enough to achieve the therapeutic objective. Therefore, conducting pre-clinical studies on EVs before advancing to the clinical phase is critical for their accurate translational applications.

## Conclusions and perspectives

Numerous recent investigations have evaluated the use of MSC-EVs in the context of regenerative therapy. These studies suggest that MSC-EVs have the ability to regulate the immune microenvironment, promote vascularization, and enhance the action, expansion, and mineral deposition of osteoblasts. Considerable headway has been accomplished in grasping the biological aspects and structural characteristics of EVs, contents, and how they affect the functions of the cells that are the focus of interaction. Indeed, it is widely recognized that both the origin of stem cells and the conditions in which they are cultured significantly impact the functional characteristics of secreted EVs. Therapies based on MSC-EVs are seen as a promising acellular therapy method, which is more manageable and minimizes the risks associated with direct cell therapy, like tumorigenesis, host rejection, and infections. Research indicates significant potential for applying EVs to enhance the effectiveness and reliability of bone and approaches aimed at regenerating periodontal tissues. However, challenges remain in achieving the optimal medical implementation of MSC-EVs, and additional research are necessary to establish an efficient procedure for designing these nano-bio elements while maintaining the precise make up and arrangement of separated EVs.

## Source of funding

This research did not receive any specific grant from funding agencies in the public, commercial, or not-for-profit sectors.

## Conflict of interest

The authors have no conflicts of interest to declare.

## Ethics approval

Not applicable.

## Consent

Not applicable.

## Authors' contributions

YA, AA, SP, and SBS drafted the main text, figures, and tables. SKH supervised the work and provided the comments and additional scientific information. LA reviewed and revised the text. All authors have critically reviewed and approved the final draft and are responsible for the content and similarity index of the manuscript.

## Availability of data and materials

Not applicable.
